# Infectious endophthalmitis leading to evisceration: spectrum of bacterial and fungal pathogens and antibacterial susceptibility profile

**DOI:** 10.1186/s12348-019-0174-y

**Published:** 2019-05-16

**Authors:** Tarjani Vivek Dave, Vivek Pravin Dave, Savitri Sharma, Roshni Karolia, Joveeta Joseph, Avinash Pathengay, Rajeev R. Pappuru, Taraprasad Das

**Affiliations:** 1 Ophthalmic Plastic Surgery Service, KallamAnji Reddy Campus, LV Prasad Eye Institute, Hyderabad, Telangana India; 2 Smt. Kanuri Santhamma Center for Vitreoretinal Diseases, KallamAnji Reddy Campus, LV Prasad Eye Institute, Hyderabad, Telangana 500034 India; 3 Jhaveri Microbiology Center, Brien Holden Eye Research Center, LV Prasad Eye Institute, Hyderabad, India; 4 Retina and Uveitis Department, GMR Varalaxmi Campus, LV Prasad Eye Institute, HanumanthawakaChowk, Visakhapatnam, Andhra Pradesh 530040 India

**Keywords:** Endophthalmitis, Evisceration, Microbiologic profile

## Abstract

**Purpose:**

To describe the spectrum of bacterial and fungal pathogens in cases of endophthalmitis requiring evisceration and report their antimicrobial susceptibilities.

**Methods:**

Retrospective, consecutive, and descriptive case series of endophthalmitis that underwent evisceration from January 2004 to December 2017. Vitreous samples from all patients had been investigated for bacteria and fungus using institutional protocol. Bacterial isolates were identified using analytical profile index (API) system until 2010 and Vitek-2 compact system (bioMérieux, France), thereafter. The susceptibility of bacterial isolates to a variety of antibiotics was determined by the Kirby-Bauer disk-diffusion method.

**Results:**

Of 791 cases reviewed, culture positivity was reported in 388 cases (48.92%). Commonest clinical setting of endophthalmitis necessitating evisceration was post-microbial keratitis (58%), followed by post-trauma and post-cataract surgery (14–15%). The commonest isolate was *Streptococcus pneumoniae*, seen in 68 samples overall (17.52%). One hundred and eighty-three isolates (47.16%) were gram-positive, 86 (22.16%) were gram-negative, and fungi constituted 137 (35.3%) isolates. *Streptococcus pneumoniae* was the commonest gram-positive bacterial isolate seen in 68/183 samples (37.15%). Among gram-negative organisms, the commonest was *Pseudomonas aeruginosa* seen in 47/86 (54.65%). *Aspergillus* spp. formed the commonest fungal isolate, 58/137 (42.33%). The susceptibility of the gram-positive bacteria was highest with vancomycin, 136/147 (92.51%) and for gram-negative bacteria was seen best with imipenem 24/29 (82.75%). Susceptibility to ceftazidime was 31/61 (50.81%) in 31/61.

**Conclusion:**

Endophthalmitis due to *Pneumococci, Aspergillus*, and *Pseudomonas* can be very fulminant and progress to require evisceration in spite of prompt and appropriate treatment.

## Summary statement

Endophthalmitis is the most severe form of intraocular infection. Due to multiple factors, sometimes the infections progress in spite of timely and appropriate management and necessitates evisceration. The current paper discusses the clinical setting, microbiologic profile, and antibiotic susceptibility pattern of cases of endophthalmitis that required evisceration.

## Introduction

Endophthalmitis is an ocular condition characterized by inflammation of the inner coats of the eye followed by exudation in the vitreous cavity [1]. It necessitates prompt and early management with intraocular antibiotics often combined with a pars plana vitrectomy. Many a times, in spite of prompt management, the condition may progress and either cause a painful blind eye or convert into a panophthalmitis with the infection spreading to the sclera and the Tenon’s capsule. In such situations, the eye often needs to undergo evisceration [2, 3]. Lu, et al. in their paper on risk factors for endophthalmitis requiring evisceration or enucleation described an evisceration rate of 14.3% [2]. Tsai, et al. in their paper on the same subject reported a higher evisceration/enucleation rate of 23.2% [3].

The probable reasons for the progression in spite of prompt and appropriate management could include relatively virulent organisms with possible high antibiotic resistance pattern. Current existing literature does not have adequate description about the spectrum of causative organisms and their antimicrobial susceptibility patterns in cases of endophthalmitis that eventually require evisceration. In the current communication, we report the above in such cases treated at our center over the past decade.

## Materials and methods

This is a retrospective, non-comparative, descriptive, consecutive case series of patients treated at L.V. Prasad Eye Institute, Hyderabad, India, both in-house and referred from January 2004 to December 2017. The microbiology records of all cases of endophthalmitis that underwent evisceration were reviewed. The study was approved by the Institutional Review Board, and it adhered to the Tenets of the Declaration of Helsinki. Vitreous samples from all patients had been investigated for bacteria and fungus using institutional protocol [4]. Bacterial isolates were identified using analytical profile index (API) system until 2010 and Vitek-2 compact system (bioMérieux, France), thereafter. The susceptibility of bacterial isolates to a variety of antibiotics was determined by the Kirby-Bauer disk-diffusion method. Fungal species were identified based on their colony and microscopic characteristics. Susceptibility test for fungal isolates were not performed.

Eviscerated samples were transported to the microbiology laboratory immediately in a sterile bottle. The sample was examined by direct microscopy (Calcofluor-white, Gram, Giemsa stains) and culture for aerobic and anaerobic organisms. Special stains such as modified Ziehl-Neelsen using 1% H_2_SO_4_ and Gomori methenamine Silver stain were done for microscopy when indicated. For culture, the sample was inoculated on 5% sheep blood agar, 5% sheep blood chocolate agar, brain heart infusion broth, thioglycollate broth, and Sabouraud dextrose agar (SDA). All media were incubated at 37 °C for 1 week except SDA, which was incubated at 27 °C for 2 weeks for the isolation of fungi. Growth on two or more media or confluent growth on at least one solid medium at the site of inoculation or growth on one medium with consistent direct microscopy result was defined as a significant positive culture and was included in the study.

## Results

A total of 6158 cases of endophthalmitis were seen at our center along the time period from January 2004 to December 2017. Of these, 791 cases of endophthalmitis underwent evisceration (12.84%). Of these cultures, positivity was reported in 388 cases (48.92%). Table 1 shows the various clinical etiologies that led to eventual evisceration. The commonest clinical setting of endophthalmitis necessitating evisceration was post-microbial keratitis which accounted for more than half (58%) of all the cases. This was followed by endophthalmitis post-trauma and post-cataract surgery (14–15%). Of the total 388 culture positive cases, the commonest isolate reported was *Streptococcus pneumoniae*, seen in 68 samples overall (17.52%). One hundred and eighty-three isolates (47.16%) were gram-positive organisms, 86 (22.16%) were gram-negative organisms, and fungi constituted 137 (35.3%) isolates. Among gram-positive organisms, *Streptococcus pneumoniae* was the commonest gram-positive bacterial isolate and the commonest gram-positive coccus seen in 68/183 samples (37.15%). The commonest gram-positive bacillus was *Bacillus* species 6/183 samples (6.55%). Among gram-negative organisms, the commonest isolate was *Pseudomonas aeruginosa* seen in 47/86 (54.65%). *Aspergillus* spp. formed the commonest isolate among fungi, 58/137 (42.33%). The detailed isolate list is described in Table 2.

**Table 1:** Etiology of endophthalmitis undergoing evisceration

Etiology	Number (%)
Post cataract surgery	55 (14.17)
Post trauma	56 (14.43)
Endogenous	22 (5.67)
Post keratoplasty	29 (7.47)
Post keratitis and perforation	226 (58.24)

**Table 2:** Spectrum of organisms isolated from the evisceration contents

	Organism	Number (%)
Gram-positive organisms (*n* = 183)	*Streptococcus pneumoniae*	68 (37.15)
	*Staphylococcus epidermidis*	31 (16.93)
	*Staphylococcus aureus*	22 (12.02)
	*Bacillus* spp.	12 (6.55)
	*Β-hemolytic Streptococci*	12 (6.55)
	*Corynebacterium* spp.	10 (5.46)
	*α- hemolytic streptococci*	7 (3.82)
	*Staphylococcus* spp.	5 (2.73)
	*Coagulase-negative staphylococci*	4 (2.18)
	*Non-hemolytic Streptococci*	3 (1.63)
	*Leuconostoc* spp.	2 (1.09)
	*Brevibacterium* spp.	2 (1.09)
	*Enteorcoccus* spp.	1 (0.54)
	*Kocuriaspp*	1 (0.54)
	*Mycobacterium* spp.	1 (0.54)
	*Nocardia* spp.	1 (0.54)
	*Pentoea* spp.	1(0.54)
Gram-negative organisms (*n* = 87)	*Pseudomonas aeruginosa*	47 (54.02)
	*Escherechia coli*	11 (12.64)
	*Enterobacter* spp.	7 (8.04)
	*Hemophilus* spp.	4 (4.59)
	*Klebsiella* spp.	4 (4.59)
	*Pseudomonas* spp.	4 (4.59)
	*Acenitobacter* spp.	3 (3.44)
	*Citrobacter* spp.	2 (2.29)
	*Serratia* spp.	2 (2.29)
	*Burkholderia* spp.	1 (1.14)
	*Weeksellavirosa*	1 (1.14)
	Non-fermenting gram-negative bacilli	1 (1.14)
Fungi (*n* = 137)	*Fusarium* spp	19 (13.86)
	Unidentified hyaline fungi	26 (18.97)
	*Aspergillus* spp.	58 (42.33)
	Unidentified dematecious fungi	11 (8.02)
	*Candida* spp.	3 (2.18)
	*Acremonium* spp.	8 (5.83)
	*Exserohilum* spp.	2 (1.45)
	*Colletotrichum* spp.	2 (1.45)
	*Phialophora* spp.	1 (0.72)
	*Alternaria* spp.	1 (0.72)
	*Penicillium* spp.	1(0.72)
	*Absidia* spp.	1(0.72)
	*Lasiodiplodia* spp.	1(0.72)
	*Curvularia* spp.	1(0.72)
	*Cladosporium* spp.	1(0.72)
	*Mucor*	1(0.72)
Parasitic organisms (*n* = 5)	*Acanthameba* spp.	5 (100)

The susceptibility of the gram-positive bacteria was highest with vancomycin, 136/147 samples tested (92.51%) followed by cefazolin118/149 samples tested (79.19%). That for gram-negative bacteria was seen best with imipenem24/29 samples tested (82.75%) followed by gatifloxacin 137/196 samples tested (69.89%), moxifloxacin101/158 samples tested (63.92%), and ofloxacin 118/199 samples tested (59.29%). Susceptibility to ceftazidime was found to be 50.81% in 31/61 samples tested. The detailed susceptibility list is described in Table 3.

**Table 3:** Overall specific antibiotic susceptibilities of the individual bacterial species^*^

Species	Cefazolin			Ceftazidime			Chloramphenicol			Ciprofloxacin			Gatifloxacin			Imipenem			Tobramycin			Vancomycin		
	S	I	R	S	I	R	S	I	R	S	I	R	S	I	R	S	I	R	S	I	R	S	I	R
Gram-positive bacteria (*n* = number tested))																								
*Streptococcus pneumoniae*	54	0	0	1	0	0	53	0	1	46	6	1	47	3	1	0	0	0	0	0	0	51	0	1
Streptococcus spp. (14)	7	0	0	1	0	0	7	0	7	3	0	3	7	0	1	0	0	0	0	1	0	7	0	0
*Staphylococcus epidermidis (23)*	19	2	1	0	0	0	19	0	4	6	4	13	18	1	4	0	0	0	0	0	0	22	0	0
Staphylococcus aureus (14)	10	2	2	0	0	0	14	0	0	1	0	13	10	2	2	0	0	0	0	0	0	13	0	0
Bacillus spp. (7)	1	0	2	0	0	1	3	0	0	7	0	0	7	0	0	0	0	0	0	0	0	7	0	0
Β-hemolytic Streptococci (6)	6	0	0	1	0	0	5	0	1	2	1	3	3	0	3	0	0	0	0	0	0	5	0	0
Corynebacterium spp. (6)	4	0	0	0	0	0	4	0	0	2	0	4	0	0	2	0	0	0	0	0	0	6	0	0
α-hemolytic streptococci (2)	0	0	2	0	0	0	0	2	0	2	0	0	2	0	0	0	0	0	0	0	0	2	0	0
Staphylococcus spp. (5)	3	0	2	0	0	0	1	0	0	0	2	3	2	1	2	0	0	0	0	0	0	4	0	1
Coagulase-negative staphylococci (4)	4	0	0	0	0	0	3	0	1	0	1	3	4	0	0	0	0	0	0	0	0	3	0	1
Non-hemolytic Streptococci (6)	1	1	0	0	0	0	1	0	1	3	0	3	1	0	1	0	0	0	0	0	0	2	0	0
Leuconostoc spp. (2)	2	0	0	0	0	0	2	0	0	2	0	0	1	0	1	0	0	0	0	0	0	2	0	0
Brevibacterium spp. (2)	1	0	1	1	0	0	2	0	0	1	0	1	1	0	1	0	0	0	0	0	0	1	0	1
Enteorcoccus spp. (1)	0	0	1	0	0	0	1	0	0	0	0	1	0	0	1	0	0	0	0	0	0	1	0	0
Kocuria spp. (1)	0	0	1	0	0	0	1	0	0	0	0	1	1	0	0	0	0	0	0	0	0	1	0	0
Mycobacterium spp.	0	0	0	0	0	0	0	0	0	0	0	0	0	0	0	0	0	0	0	0	0	0	0	0
Nocardia spp. (1)	0	0	1	0	0	1	0	1	0	0	1	0	0	1	0	0	0	0	0	0	0	1	0	0
Pentoea spp. (1)	0	0	0	0	0	1	1	0	0	0	1	0	0	1	0	0	0	0	1	0	0	0	0	0
Gram-negative bacteria																								
*Pseudomonas aeruginosa (33)*	0	0	7	14	2	14	2	2	29	17	0	16	15	1	17	17	0	3	6	0	10	0	0	6
Escherechia coli (6)	0	0	0	2	0	4	6	0	0	2	0	4	2	1	3	3	0	0	1	0	2	0	0	0
Enterobacter spp. (2)	1	0	0	1	0	1	2	0	0	2	0	0	2	0	0	1	0	0	1	0	0	1	0	0
Hemophilus spp. (2)	0	0	0	1	0	0	2	0	0	0	0	1	2	0	0	1	0	0	1	0	0	0	0	0
Klebsiella spp. (2)	0	0	0	1	0	1	0	0	0	1	0	1	1	0	1	0	1	0	1	0	0	0	0	0
Pseudomonas spp. (4)	0	0	1	3	0	1	1	0	3	0	0	4	2	0	1	0	0	1	1	0	1	0	0	1
Acenitobacter spp. (0)	0	0	0	0	0	0	0	0	0	0	0	0	0	0	0	0	0	0	0	0	0	0	0	0
Citrobacter spp. (1)	0	0	0	1	0	0	1	0	0	1	0	0	1	0	0	0	0	0	0	0	0	0	0	0
Serratia spp. (1)	0	0	0	1	0	0	1	0	0	0	0	1	1	0	0	0	0	0	1	0	0	0	0	0
Burkholderia spp. (1)	0	0	0	0	0	1	0	0	1	0	0	1	0	0	1	1	0	0	0	0	1	0	0	0
Non-fermenting gram-negative bacilli (1)	0	0	0	1	0	0	1	0	0	1	0	0	1	0	0	0	0	0	0	0	0	0	0	0
Weeksellavirosa (1)	1	0	0	0	0	0	1	0	0	1	0	0	1	0	0	0	0	0	0	0	0	1	0	0
Total	123	5	20	29	2	25	134	5	48	100	16	77	132	11	42	23	1	4	13	1	14	130	0	11

*Not all samples were tested for all antibiotics

## Discussion

The current study showed that the commonest isolate in cases of endophthalmitis undergoing evisceration was *Streptococcus pneumoniae* followed by *Aspergillus* and *Pseudomonas aeruginosa* respectively. Only one previous study on evisceration following endophthalmitis has discussed the causative organisms [3]. In that study, the authors described 20 eyes that underwent evisceration following endophthalmitis. The commonest reported organism in that subset was *Pseudomonas aeruginosa*. *Aspergillus* accounted for one eye whereas *Streptococcus pneumoniae* was not reported. Studies on endophthalmitis secondary to *Streptococcus pneumoniae* suggest a poor visual outcome with a high rate of poor anatomic outcome. Miller et al., in their study on pneumococcal endophthalmitis, reported 3/27 (11.11%) eyes needing evisceration [5].The low evisceration rate in their study could be attributed to the fact that the antimicrobial susceptibilities to the commonly used intravitreal antibiotics like vancomycin, gatifloxacin, cefazolin, and ciprofloxacin, in their study was 100%. Conversely, in the current study, the pneumococcal endophthalmitis subgroup (68 eyes) showed a varied sensitivity pattern to the common antibiotics used. In contrast to Miller et al., in another study by Soriano et al. [6], the evisceration rates in 36 cases of *Streptococcus pneumoniae* endophthalmitis was 47.22%. This indirectly is in agreement with our study observation that *Streptococcus pneumoniae* is an important cause of evisceration following endophthalmitis. This relatively poor outcome has been hypothesized due to a very high degree of inflammatory response evoked by *Streptococcus pneumoniae* by its exotoxins and enzymes [7–10]. A commonly known virulence factor is a polysaccharide capsule that releases pneumococci from the host by preventing phagocytosis. Another potent virulence factor is pneumolysin which inhibits host responses such as antibody synthesis and lymphocyte proliferation. Inflammation is caused by the virulence factor of cell wall components, which are thought to be the main cause of symptoms. *Aspergillus* is a common organism causing endophthalmitis, more so in the Indian sub-continent as compared to the western world, where molds like *Candida* form the major etiology of fungal endophthalmitis [11–13]. In cases of fungal endophthalmitis especially with filamentous fungi, evisceration or enucleation rates as high as 25% have been reported [12, 13]. Occurrence in immuno-competent individuals common in the Indian sub-continent and a low index of suspicion initially often leads to a delay in the diagnosis of fungal endophthalmitis. This delay especially in case of *Aspergillus* like fungi can cause widespread vascular spread and intraocular tissue necrosis causing loss of anatomic integrity and necessitating evisceration [14].

EIfrig et al [15] in their study on *Pseudomonas* endophthalmitis reported an evisceration/enucleation rate of 64%. Similarly, high evisceration rates post *Pseudomonas* endophthalmitis were also reported by other workers [16, 17].This is attributable to the widespread and rapid tissue necrosis caused by *Pseudomonas* toxins. These toxins are known to disrupt cellular membranes and epithelial barriers and cause cytotoxicity [18, 19].

The current study specifically looked at the microbiologic profile and antibiotic susceptibility pattern of bacteria associated with endophthalmitis that underwent evisceration. The study also shows that the commonest predisposing clinical setting that culminates into need for evisceration is endophthalmitis following keratitis and perforated corneal ulcer. Other than the microbiologic profile, various other clinical factors like duration of the infection, etiology of endophthalmitis, type of treatment, associated trauma, and comorbid systemic factors may have a role in the final outcome, but the current study was not designed to look at those factors. In conclusion, the current communication suggests that though traditionally speaking gram-positive bacterial endophthalmitis has a relatively better treatment outcome as compared to gram-negative endophthalmitis, in cases progressing the evisceration, the incidence of *Streptococcus pneumonia*, *Pseudomonas*, and *Aspergillus* was very high.
